# Supporting teams to optimize function and independence in Veterans: a multi-study program and mixed methods protocol

**DOI:** 10.1186/s13012-018-0748-3

**Published:** 2018-04-20

**Authors:** Virginia Wang, Kelli Allen, Courtney H. Van Houtven, Cynthia Coffman, Nina Sperber, Elizabeth P. Mahanna, Cathleen Colón-Emeric, Helen Hoenig, George L. Jackson, Teresa M. Damush, Erika Price, Susan N. Hastings

**Affiliations:** 1 Health Services Research and Development Center of Innovation, Durham Veterans Affairs Health Care System, 508 Fulton St., Durham, NC 27705 USA; 2 Department of Population Health Sciences, Duke University School of Medicine, Durham, NC USA; 3 Department of Medicine, Duke University School of Medicine, Durham, NC USA; 4 Department of Medicine and Thurston Arthritis Research Center, University of North Carolina at Chapel Hill, Chapel Hill, NC USA; 5 Department of Biostatistics and Bioinformatics, Duke University School of Medicine, Durham, NC USA; 6 Geriatric Research Education and Clinical Center, Durham VA Health Care System, Durham, NC USA; 7 Physical Medicine and Rehabilitation Service, Durham VA Health Care System, Durham, NC USA; 8 Health Services Research and Development Center for Health Information and Communication, Roudebush Veterans Affairs Medical Center, 1481 W. 10th St., HSRD 11H, Indianapolis, IN 46202 USA; 9 Department of General Internal Medicine and Geriatrics, Indiana University School of Medicine, Indianapolis, IN USA; 10 Regenstrief Institute, Inc., Indianapolis, IN USA; 11 San Francisco VA Care System, 94121, 4150 Celement St., Box 111, San Francisco, CA USA; 12 Department of Medicine, University of California San Francisco, San Francisco, CA USA

**Keywords:** Implementation, Function, Veterans, VA, Healthcare team

## Abstract

**Background:**

Successful implementation of new clinical programs depends on effectively establishing, reorganizing, or enhancing team structures and processes to coordinate the work of individuals who are interdependent in their tasks, manage relationships, and share responsibility for outcomes. However, a one-size-fits-all approach is rarely effective. In partnership with VA national clinical leaders and local clinical champions, the Optimizing Function and Independence VA Quality Enhancement Research Initiative program (Function QUERI) will evaluate efforts to implement team-based clinical programs for Veterans at risk for functional decline and disability.

**Methods:**

Function QUERI will implement and evaluate three innovative, evidence-based clinical programs in VA medical centers: (1) a group physical therapy program for knee osteoarthritis (Group PT); (2) assisted early mobility for hospitalized older veterans (STRIDE), a supervised walking program for hospitalized older veterans; and (3) implementation of helping invested family members improve veteran experiences study (iHI-FIVES), a skills training program for caregivers of disabled Veterans. A common reason for clinical care gaps in these populations is poor communication and coordination among the many interdisciplinary providers involved in their care. To facilitate the implementation of the clinical programs, Function QUERI will evaluate the impact of complexity science-based implementation intervention to promote team readiness (CONNECT), an implementation intervention designed as a bundle of interaction-oriented activities to promote team function and readiness for change, on the implementation of clinical programs across multiple sites. The evaluation will use a mixed methods design. Group PT is a local, single-site quality improvement project where a modified CONNECT intervention will be tested to inform the remaining program implementation projects. For STRIDE and iHI-FIVES projects, we will randomize participating sites to implement the clinical program, with the CONNECT intervention or not, and will use a stepped-wedge cluster randomized trial design.

**Discussion:**

Function QUERI will translate its findings across its projects to identify the contextual factors and components from CONNECT that improve team processes and function to optimize effective implementation for future rollout of VA clinical programs. Synthesizing findings within and across projects, we will specify dimensions of team characteristics and function that enhance capacity for clinical innovation and uptake of evidence-based programs.

**Trial registration:**

NCT03300336 Registered September 28, 2017, NCT03474380 Registered March 15, 2018.

**Electronic supplementary material:**

The online version of this article (10.1186/s13012-018-0748-3) contains supplementary material, which is available to authorized users.

## Background

Clinical improvement efforts require team structure that coordinates the work of individuals who are interdependent in their tasks, manage relationships within one or more social systems (e.g., service line, facility), and share responsibility for outcomes. Teams influence relationship patterns that emerge within workgroups [1], which have a significant impact on individual and collective attitudes, behaviors, and performance [2, 3]. While most organizational change and quality improvement (QI) efforts focus on changing individual behavior, less attention has been paid to improving the work and processes of teams. Effective management strategies that focus on fostering productive interdependencies among individuals engaged in change efforts may lead to better outcomes than strategies focusing on individuals alone [4, 5].

Team factors are especially important in interdisciplinary care settings where expertise and skills vary among clinicians involved in patient care. Moreover, there is limited evidence elucidating the features of teams (i.e., size, communication, role clarity) that are associated with clinical performance and successful implementation of non-acute services in hospital and specialty care settings. Consistent with an emerging literature indicating that team process and effectiveness are associated with improvements in patient-centered care [6], the Optimizing Function and Independence VA Quality Enhancement Research Initiative (QUERI) program (hereafter Function QUERI) will evaluate the impact of teams on the implementation and effectiveness of new clinical programs in specialized and acute care settings for patients at risk for impairment of function and independence. In addition, Function QUERI will test an intervention designed to improve team function and readiness for change, as a strategy for promoting effective implementation of Function QUERI’s clinical programs.

To accomplish Function QUERI’s overall goals, we will:

1. Implement and evaluate three new clinical programs, *Group PT* for knee osteoarthritis, *STRIDE*, and *iHI-FIVES*, that fill gaps in current clinical care for Veterans at risk for functional decline and disability
2. Adapt a novel complexity science-based implementation intervention to promote team readiness (*CONNECT*) for use in a diverse mix of clinical settings and Veterans Affairs Medical Centers (VAMCs)
3. Examine the impact of CONNECT and team processes on implementation within and across projects.

## Methods

### Overview of the three program projects

The Function QUERI program will implement and evaluate three innovative, evidence-based clinical programs that fill gaps in current clinical care for Veterans at risk for functional decline and disability and will be conducted in VA medical centers throughout the national VA Health Care System. Guided by the International Classification of Functioning, Disability and Health [7], Function QUERI’s clinical programs directly address known stressors and contextual factors that influence functional ability and independence. The three clinical programs will be implemented as QI projects, and the evaluation will be conducted as human subjects research approved by the Durham VA Health Care System Institutional Review Board.

The first of the three Function QUERI projects evaluates the implementation and features of Group PT, a group-based physical therapy program to improve access to therapy for Veterans with knee osteoarthritis (OA). With a lifetime risk of 45% and rising overall prevalence [8, 9], knee OA is one of the most common chronic health conditions and a leading cause of pain and disability among adults [10–16]. Despite evidence that PT improves knee OA pain, disability, and other key outcomes [17–22], PT is underutilized [23]. Group PT is designed to enhance efficiency of care by providing more contact hours of care per patient with fewer total clinician hours, compared to traditional individual PT. The program involves six 1-h, weekly, group-based sessions comprising up to 10 patient participants. At patients’ first session, a physical therapist conducts a brief evaluation and administers baseline functional tests and questionnaires; functional tests and questionnaires are repeated at the last session. At each session, the physical therapist leads the group in strengthening and stretching exercises, as well as brief education and discussion modules. Patients are instructed to perform assigned exercises at home. Results from a trial demonstrated similar outcomes between Group PT and traditional individual PT for knee OA, but the Group PT program has a lower cost [24]. Group PT is a 1-year project that takes place in a single site to evaluate key outcomes of sustainability and ways to improve the reach of the program for wider dissemination. It also serves as a case study for the CONNECT implementation intervention, the experiences of which will inform refinements to CONNECT trainings and content for application in other Function QUERI projects.

Project 2 evaluates the implementation of assisted early mobility for hospitalized older veterans (STRIDE), a supervised walking program for hospitalized older Veterans focused on maintaining musculoskeletal strength and mobility during hospitalization, a highly vulnerable time for development of disability. A key contributor to hospital-associated disability is immobility during hospitalization [25]. While fewer than 5% of patients have physician orders for bed rest, hospitalized older adults spend only 4% of their time standing or walking [26]. The hazards of immobility in the hospital have been recognized for more than 2 decades [27]. Previously developed hospital mobility interventions have demonstrated the potential of inpatient walking to prevent declines in mobility during hospital stays and to reduce hospital lengths of stay [28–34], but there are currently no VA system-wide approaches to address this important gap in clinical care. Adapted from a mobility program tested in three non-VA hospitals that led to reduced hospital lengths of stay [35], STRIDE is designed for patients aged > 60 and consists of a one-time gait and balance assessment conducted by a physical therapist, followed by daily supervised walks by a recreation therapy assistant for the duration of the hospital stay [36]. Clinical demonstration of STRIDE conducted at the Durham VA Health Care System (VAHCS) resulted in a greater likelihood of discharge to home (than to skilled nursing or rehabilitation) among STRIDE participants compared to clinically similar patients receiving usual care (92 vs 74%, *p* = 0.007) [36]. Based on the cumulative evidence on early mobility, positive staff, and patient assessments of STRIDE, it was established as a permanent clinical service at the Durham VAHCS and has the potential to become a system-wide approach to address hospital-associated disability in the VHA. Function QUERI’s STRIDE implementation is a multi-year project that will be implemented across eight participating VAMCs.

Project 3 examines the implementation of helping invested family members improve veterans experiences study (iHI-FIVES) to promote function and independence through skills training and support for caregivers of Veterans with cognitive and/or functional limitations. The Veterans Health Administration (VHA) has the most extensive system of home and community-based services of any health care system in the USA and yet about two-thirds of the 5.5 million Veterans who receive care in the home receive it exclusively from family and friends. Caregivers allow Veterans to avoid or delay nursing home entry [37], but caregiving can result in high rates of caregiver burden, depression, cost, and health risk to Veterans themselves. Caregivers commonly report unmet needs for caregiver skills training [38], which can reduce negative consequences of caregiving, increase quality of care, and optimize patient independence [39–44]. HI-FIVES is a multi-modal training program that occurs after a Veteran’s referral to home and community-based services. Its training sessions address standardized and caregiver-selected topics such as increasing Veteran function and independence, caregiver injury prevention and self-care, communicating with providers, and navigating the VHA [45]. Caregivers are encouraged to create action items to apply the skills learned, including incorporating the use of relaxation techniques into their busy lives. Results from the HI-FIVES trial (NCT01777490) found that HI-FIVES significantly increased Veteran and caregiver’s experience of VA care. The trial yielded limited effects on increasing patient days at home, which may be explained by the trial’s limitation of being underpowered to detect statistically significant differences (due to larger than expected variance). Thus, a larger sample size is needed to detect true differences in days at home, the primary outcome. Based on the results to date, the VA National Program Office on Caregiver Support is promoting HI-FIVES for wide-scale dissemination. Function QUERI will capitalize on this effort and support the implementation of HI-FIVES (iHI-FIVES*)* project by facilitating the rollout of caregiver trainings across eight participating VAMC sites and evaluating the program, with its increased enrollment for definitive retesting on a larger sample (*n* ≥ 400) in this hybrid type III effectiveness-implementation design. Caregiver Support Program staff and Geriatrics and Extended Care Services staff will be recruited to offer iHI-FIVES.

As described in Table 1, Function QUERI’s three clinical programs promote a shared goal of optimizing physical function and independence among Veterans while potentially delaying the onset of costly disability. An important challenge and consideration is understanding optimal methods to disseminate these promising programs more widely and in a sustained way. Our initial program experience suggests that inter-professional relationships and team dynamics are the key determinants to the success of new hospital-based clinical programs that require collaborative processes involving multiple disciplines. Indeed, a major reason for clinical care gaps in this population is poor communication and coordination among the many interdisciplinary providers involved in their care. Implementing new programs in diverse settings requires buy-in and cooperation among many different service lines, and a one-size-fits-all approach is rarely effective.

**Table 1:** Overview of optimizing function and independence QUERI (Function QUERI) projects

Overview of projects	Group PT	STRIDE	iHI-FIVES
	Outpatient physical therapy	Inpatient walking assistance	Caregiver (CG) skills training
Context of clinical program			
Relevant clinical service	Physical medicine and rehabilitation (PM&R)	Inpatient general medicine	Caregiver (CG) support program or other service lines that support CGs
Nature of clinical program tasks	Existing task, alternative mode of delivery	New task	Existing task, alternative mode of delivery
Team formation	Existing team (processes)	Existing team (formation) New team (processes)	New team (formation) Existing team (processes)
Program delivery: boundary spanning?	No	Yes: general medicine, nursing, physical therapy	Both (across service lines, within CSP or GRECC)
Team membership: roles in program delivery	• Physicians: referral • Physical Therapist: initial evaluation, lead sessions • Physical Therapy Assistant: co-lead sessions	• Physicians: referral • Registered nurse: coordination, assessment • Physical therapist: assessment, walking, supervise walks • Nurse assistant, physical therapy assistant, volunteer: supervise walks	• Physicians: referral • Social worker: referral and training • CG support coordinator: training • Registered nurse: training • Psychologist: training
Noted challenges in program delivery to be addressed by CONNECT	• Awareness of Group PT outside PM&R • Clarity of roles • Established procedures • Interaction within the PM&R service	• Clarity of roles • Availability of staff resources • Communicating relevant clinical information and “prescribed dose” of STRIDE	• Awareness of the CSP and its mission in clinical units • Clarity of roles, fragmented/duplicative services for CG support across service lines, enhance continuity of caregiver support
Outcomes of interest			
Implementation outcomes	✓ Penetration ✓ Fidelity ✓ Provider experience	✓ Penetration ✓ Fidelity ✓ Team processes ✓ Provider experience	✓ Penetration ✓ Fidelity ✓ Team processes ✓ Provider experience
Patient/service-level outcomes	✓ Function ✓ Pain ✓ Wait times ✓ Patient satisfaction ✓ Provider satisfaction ✓ Budget impact	✓ Discharge to SNF ✓ Physical function ✓ Community mobility ✓ HRQoL, sleep, depression ✓ Hospital LOS ✓ Patient satisfaction ✓ Budget impact	✓ Days in community ✓ CG Depressive symptoms ✓ CG Burden ✓ CG Satisfaction with VA care received ✓ Budget impact

### Implementation core

Providing expertise in implementation science, organizational behavior, and quantitative and qualitative analyses, our implementation core serves as an essential bridge across all Function QUERI projects. The implementation facilitation component of the core will develop and facilitate the implementation activities for Group PT, STRIDE, and iHI-FIVES with participating sites. The data and analytic component of the core will develop survey and interview instruments, establish common measurement of team characteristics and outcomes across projects (when possible), and evaluate the impact of teams on implementation and programmatic outcomes. Organizing the projects through this implementation core provides economies of scale and minimizes variation in processes and effort across projects and the participating sites. Implementation core activities are described in more detail below.

### Guiding framework for implementation and evaluation

Our program of work is informed by Grol and Wensing’s Implementation of Change Model (IoC) [46] and the Consolidated Framework for Implementation Research (CFIR) [46, 47] which, in combination, provide a comprehensive approach to implementing new clinical programs, from development through execution, evaluation, and dissemination. Specifically, the IoC model describes a process of implementation, beginning with the identification of practices (i.e., clinical programs) to address gaps in care for patients with (or at-risk for) impaired function and independence, defining targets for improvement, developing and executing implementation strategies, and continuous evaluation and adaptation of implementation to improve processes and outcomes. IoC is consistent with tenets of complexity science, acknowledging that implementation processes in complex systems require flexible and iterative approaches and constant monitoring and assessment [46, 48]; IoC is thus an appropriate framework to guide the development and execution of Function QUERI’s implementation strategies (described in more detail below). Evaluation and dissemination approaches are informed by the CFIR, which is a macro-level model of implementation that describes a comprehensive taxonomy of operationally defined domains influencing implementation. Each CFIR domain is informed by an array of theoretical constructs from various disciplines. We adapted CFIR to inform our evaluation of team-based implementation strategies for Function QUERI’s complex clinical programs.

**Fig. 1:**
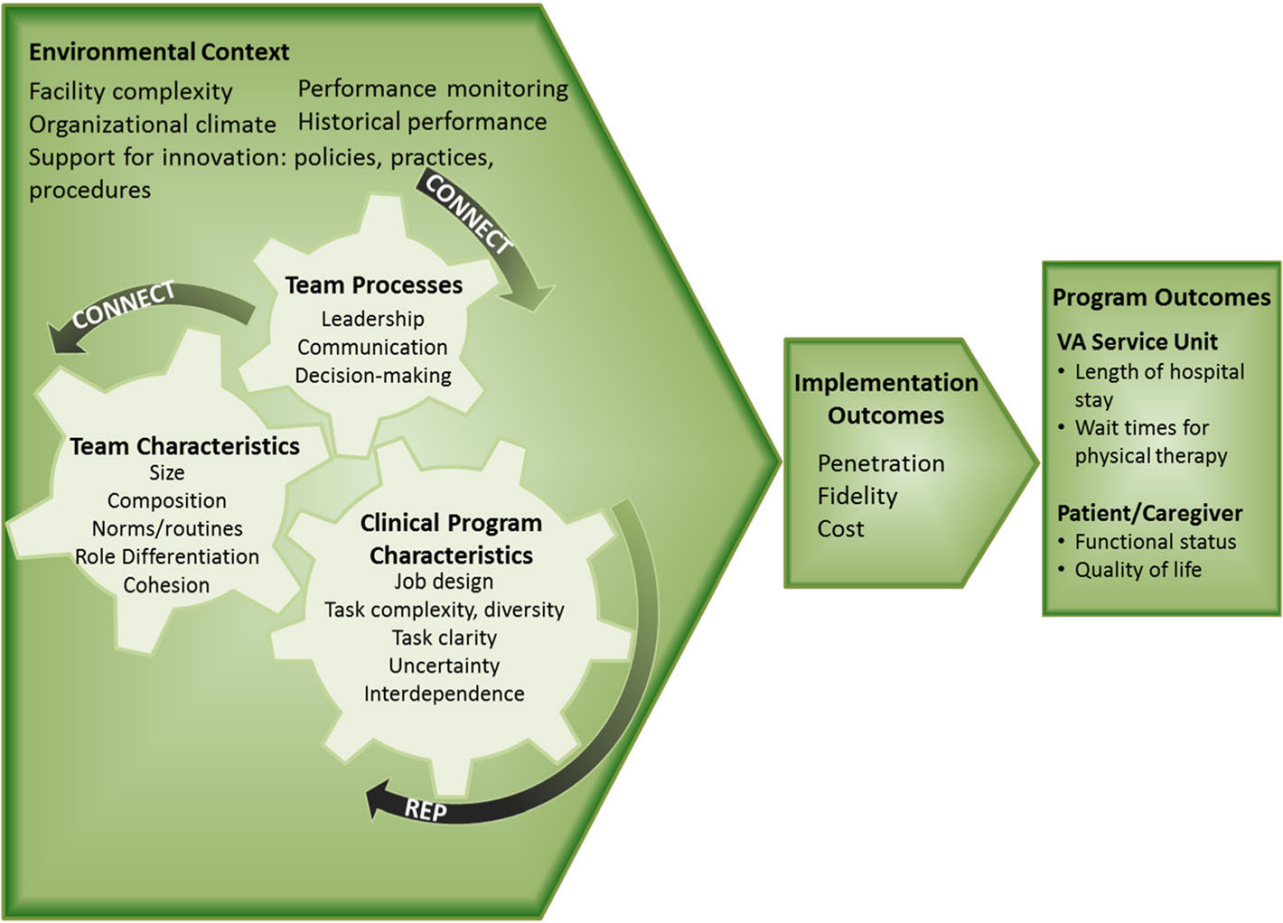
Nested model of team function and performance in implementation. Notes: (1) Team characteristics and processes, their environmental context, and the prescribed work characteristics of Function QUERI’s clinical programs both affect and are affected by one another in a nonlinear fashion. Collectively, these factors are critical to successful implementation of new clinical programs and improvements in patient care. (2) Replicating Effective Programs (REP) is a framework of processes for implementing new clinical programs into VA practice. Function QUERI REP processes, by project, are described in Additional file 1. (3) CONNECT is a novel implementation intervention to facilitate and enhance team function and readiness to implement Function QUERI’s new clinical programs

We use our program’s nested model of team function and performance (Fig. 1) to guide our evaluation of implementation, which draws from complexity science [4, 48], organizational theories on change and team behaviors, and the CFIR. Our model posits that the successful implementation of clinical programs is a function of team structure, team processes (i.e., CFIR’s inner setting), the environmental context (i.e., outer setting), and the prescribed work characteristics of our innovative clinical programs. Altogether, these factors affect providers’ engagement, interactions, and work activities, which collectively affect the ability of teams to accomplish their goals. Understanding the relationships among clinical program characteristics, team processes, team characteristics, and environmental contexts is critical to implementing and evaluating programs for Veterans at risk for functional decline and loss of independence.

### Implementation strategies: activities and intervention

#### Overview

Based on preliminary assessment of barriers to program implementation for each project (noted in Table 1) and input from our clinical and operational VA partners, Function QUERI will use Replicating Effective Programs (REP) as the overarching implementation framework for incorporating new clinical programs into routine practice [49]. REP has many advantages for implementation; for Function QUERI, REP is ideal because it provides a structure for specifying core elements of a program to be disseminated and for operationalizing elements that can be adapted to local settings. However, there are limitations to its use as well. In particular, an often noted barrier to implementation of new clinical programs is a focus on the clinical program content while ignoring the organizational learning context and processes needed to successfully implement change [50]. Function QUERI addresses this challenge by testing an implementation strategy designed to improve team function and readiness for change, which we posit as a pre-condition of effective implementation of clinical programs involving interdisciplinary care teams. In concert with program implementation, the implementation core will pair REP, the implementation framework, with an innovative implementation intervention—CONNECT—to facilitate the readiness of teams to adopt new clinical programs [50, 51].

#### Replicating Effective Programs

Replicating Effective Programs (REP) is a package of strategies, originally developed to support dissemination of behavioral and treatment interventions for HIV in community settings [52]. REP has been described both as a framework for implementation processes [49] and as a strategy that has been effective in implementing new practices [53, 54]. Function QUERI uses REP as a framework, or guide for implementation, which comprises an integrated bundle or package of discrete, standardized activities that address common barriers to implementation success [54, 55]. REP is designed for rollout of new programs through four phases of activity: pre-condition, pre-implementation, implementation, and maintenance. Across these phases, REP is delivered through a combination of standardized activities (Additional file 1). Each Function QUERI project has already initiated the REP pre-condition phase, by identifying needs and gaps in clinical care and the clinical programs that will be used to fill these gaps. Pre-implementation phase activities will include drafting clinical program implementation packages, with input from stakeholders from VA operations. Packages include standardized program materials for clinical staff to implement the program (e.g., training manuals, procedures, competencies) and guidance on core elements of the program and options for customization. Throughout the implementation and maintenance phases, Function QUERI will also provide technical assistance and support to participating sites. The final step in REP is refining clinical program implementation packages in preparation for wider-scale dissemination.

#### CONNECT

Informed by social constructivist and learning theories and complexity science, which describe learning as a social process drawn from engaged interactions and information flow, CONNECT is a bundle of interaction-oriented activities designed to supplement implementation efforts by promoting team function and readiness for change [50, 56–61]. CONNECT was originally developed for fall prevention in nursing homes and has been associated with improvements in communication and participation in decision-making among clinical staff, as well as resident outcomes [51, 62, 63]. As with REP, CONNECT’s design is suitable for rollout in various contexts and phases of implementation. For example, CONNECT may be an essential process for the creation of functional relationship networks and communication channels for learning, information exchange and problem solving for new clinical teams or programs in VAMCs (e.g., STRIDE), or as an effective team “booster” for existing groups that are incorporating new functions in clinical programs (e.g., Group PT, iHI-FIVES). CONNECT activities include group-based sessions designed to increase connections and information flow between providers and encourage them to seek out alternative explanations from others to make sense of new clinical data (cognitive diversity). Facilitator-led, group sessions use storytelling and role play to practice new behaviors. Additional sessions involve individuals mapping their relationships and communication patterns, discussing strategies for creative problem solving, and individual mentorship to sustain new interaction behaviors [51]. Function QUERI will evaluate the extent to which CONNECT affects team processes and characteristics, to facilitate team function (i.e., CFIR’s inner setting) and in turn to affect implementation, patient, and service outcomes.

Function QUERI will adapt the CONNECT intervention for application in Function QUERI clinical contexts, where new clinical programs are composed of team members from multiple service units within a VA hospital. For example, participants targeted for CONNECT training vary across the three clinical program’s delivery teams and their referring providers. CONNECT for the two larger, longer term projects will be informed by initial work of the single-site Group PT project, which will identify relevant components of CONNECT for improving team communication. Furthermore, CONNECT activities and didactic content will be modified to fit each clinical program. Role play will include scenarios related to issues surrounding each project’s clinical problem, and the emphasis on CONNECT activities may differ. To illustrate, activities known as group and individual mapping are the likely focus of CONNECT team building for Group PT and iHI-FIVES because team members of these clinical programs work in different locations (i.e., service units) with limited opportunities for frequent interaction and thus require support for improving communications about appropriate referrals, administrative logistics, and communicating patients’ clinical status. CONNECT training for STRIDE and iHI-FIVES will be conducted prior to launch of clinical programs across multiple sites.

### Study design

The Function QUERI’s program goals are to assess (1) the effectiveness of the clinical programs and (2) the impact of CONNECT and team characteristics on clinical program implementation. The remainder of this paper focuses on the design of our evaluation of teams and CONNECT. Details on evaluations of clinical program effectiveness (i.e., patient outcomes) are available at clinicaltrials.gov (STRIDE no. NCT03300336 and iHI-FIVES no. NCT03474380).

#### Project 1: Group PT

As a 1-year quality improvement project at a single site, the study design for Group PT is different than that of our multi-site implementation projects. Because the Durham VA Health Care System’s Physical Medicine and Rehabilitation Service had already initiated implementation of Group PT for Veterans with knee OA, Function QUERI’s role is to evaluate the program’s successes, challenges, and outcomes to inform programmatic adaptations in a rapid timeframe, akin to a Plan-Do-Study-Act cycle [64–66]. Function QUERI will also adapt and provide CONNECT Group-to-Group relationship mapping training to the team delivering Group PT, specifically tailored to address our operational partner’s goals (Physical Medicine and Rehabilitation at the Durham VA Health Care System) to improve connections with providers from other service units and enhance communication about referrals and scheduling logistics. Using a mixed method design, we will continuously evaluate the Group PT program and identify influences of CONNECT on program outcomes, using quantitative and qualitative data reported quarterly to inform any refinements for the next 3-month implementation cycle. Importantly, implementation processes and evaluation from Group PT will be used to inform adjustments of CONNECT for our implementation of STRIDE and iHI-FIVES at multiple participating VAMCs.

#### Projects 2 and 3: STRIDE and iHI-FIVES

Clinical program implementation and testing of the CONNECT intervention for the STRIDE and iHI-FIVES implementation projects use a hybrid type III effectiveness-implementation design because the implementation intervention (CONNECT) and clinical programs (STRIDE and iHI-FIVES) have solid evidence bases but could yield weaker outcomes in new or less controlled environments [51, 67, 68]. To evaluate the effect of the clinical programs on Veteran outcomes, both projects use a stepped-wedge cluster randomized trial (CRT) design, which is ideal when the benefits of clinical programs are known but it may be logistically implausible to roll out the intervention simultaneously to all participating sites [69].

**Fig. 2:**
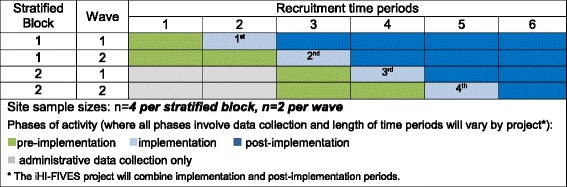
Stepped-wedge design for the eight participating sites for STRIDE and iHI-FIVES implementation projects. Site sample sizes: *n* = *4* per stratified block, *n = 2* per wave*.* Phases of activity (where all phases involve data collection and length of time periods will vary by project*): ■pre-implementation, ■implementation, ■ post-implementation,.and ■ administrative data collection only. *The iHI-FIVES project will combine implementation and post-implementation periods

STRIDE and iHI-FIVES will each recruit eight unique participating sites for implementation and evaluation. Each project’s implementation will occur in two stratified blocks (four VAMCs per block) with two waves per block (two VAMCs per wave) (Fig. 2). We will use two waves to minimize risk of site attrition based on long waits between enrollment and assigned implementation start date. Within blocks, each wave consisting of two VAMCs is randomized to a time period for implementation rollout (e.g., period 2 or 3 for sites in block 1 in Fig. 2). The length of the time periods may vary between projects (e.g., 3 or 6 months). Then, within each wave, each VAMC will be randomized to receive implementation consisting of either REP (standalone) or REP + CONNECT, with randomization assigned by a random number generated and conducted by study statisticians. Within the stepped-wedge design, there will be six time periods per VAMC site that are differentially split between pre-implementation (control) and post-implementation (treatment) periods depending on the wave and block of a site (see Fig. 2).

### Participating sites

With assistance from our VA operation partners and marketing clinical programs through VAMC clinical conferences and national meetings, we will recruit sites on a volunteer basis. We will assess eligibility of sites to determine if they meet project-specific criteria that includes adequate sample size for evaluation. Specifically, STRIDE sites must have a minimum inpatient average daily census of 20 general medicine patients per day and iHI-FIVES sites must commit to conducting two rounds of iHI-FIVES training sessions every 6 months.

### Data: measurement and sources

Evaluation of team and CONNECT impacts on implementation will employ a mixed methods design. Guided by our nested model of team function and performance in implementation, the implementation core will develop and collect common measures from primary and secondary data sources to evaluate team processes and characteristics, program characteristics, environmental context, and implementation outcomes. The data used to evaluate team and CONNECT impacts on clinical program implementation will be obtained from several sources, including provider/staff surveys and interviews, patient surveys and interviews, patient electronic medical record abstraction, and VA administrative data for patient encounters and VAMC performance measures. Since all projects’ clinical programs utilize staff from existing care teams, inpatient wards, and service lines, Function QUERI will assess team characteristics and function before and after CONNECT training and program implementation. Sample measures and associated data sources are displayed in Table 2.

**Table 2:** Function QUERI measures and data sources

Data element	Data sources				Projects
	VA admin	Survey	Interview	Field notes	
Team characteristics					
Team size: number of members of team for clinical program delivery			X		1, 2, 3
Team composition: diversity of members within team to accomplish tasks (e.g., expertise and skill set) and boundary spanning among team memberships		X	X		1, 2, 3
Role clarity: extent to which roles among team members are clearly defined		X	X		1, 2, 3
Cohesion: commitment in working as a collective unit to accomplish the work of the team		X	X		1, 2, 3
Team processes					
Communication: open communication and participation in handling conflict and solving problems as a collective unit		X	X		1, 2, 3
Communication structure/channels: standardization and centralization of conveying key information within team and externally			X		1, 2, 3
Goal and means clarity: collective understanding of the work of the team and its goals, and agreement on how their goals are reached		X	X		1, 2, 3
Decision-making: manner in which information is exchanged and decisions are made within teams (e.g., member involvement, techniques of decision-making)		X	X		1, 2, 3
Task interdependence: degree of dependence of tasks between \members within a team (team- and individual-level measures)			X		1, 2, 3
Satisfaction/experience: employee satisfaction with the outcomes of the team’s work, to date.		X	X		1, 2, 3
Clinical program/task characteristics—Group PT, STRIDE, and HI-FIVES					
Task uncertainty: predictability in the work processes; presence of standardized processes and protocols for different clinical scenarios			X		1, 2, 3
Program interdependence: degree of dependence of tasks on other clinical units		X	X		1, 2, 3
Task interdependence: degree of dependence of tasks between team members		X	X		1, 2, 3
Environmental context					
Facility complexity: operational complexity of VAMC (e.g., patients served, case-mix, and intensive care unit level)	VA Planning				1, 2, 3
Climate: share perception on the degree to which clinical program is supported, rewarded, and expected within VAMC		X	X		1, 2, 3
Leadership, clinical champion			X		
Policies, practices, and procedures: organizational effort to support innovative practices within VAMC (e.g., performance monitoring)			X		1, 2, 3
Historical performance: prior innovation history, organizational performance on related clinical metrics	SAIL, IPEC, AES		X		1, 2, 3
Implementation outcomes					
Penetration: reach (referrals, initiation rate), integration of program within VAMC’s relevant clinical units	Chart review VA visits, claims	X	X	X	1, 2, 3
Fidelity: degree to which program is implemented • Adherence to protocol • Participant engagement	Project records Chart review VA visits, claims		X	X	1, 2, 3
Cost • Total implementation cost • Total program delivery cost • Resource utilization costs	Project records VHA salary		X	X	2, 3
Clinical and service-level outcomes (sample measures obtained at the patient-level)					
Program service use: referrals, scheduled appointments, participation, attendance, distance walked,	Chart review VA visits, claims				
Function: function and disability instrument, WOMAC pain and physical function scale, Zarit subjective burden scale, Center for Epidemiology Studies Depression Scale	Chart review	X			1, 2, 3
Independence: days in home/community, discharge to nursing home, skilled nursing, wait times					2, 3
Quality of life: health-related quality of life		X			
Resource utilization: outpatient visits, hospitalization, ER, discharge to nursing home, skilled nursing, wait times	Chart review VA visits, claims				1, 2, 3
Patient satisfaction: CAHPS survey items on satisfaction		X	X		1, 2, 3

*Notes:* Project 1: Group PT (single site, 1 year); project 2: STRIDE (eight sites, multi-year); project 3: iHI-FIVES (8 sites, multi-year)

*Team characteristics and processes* will be primarily assessed using the Team Development Measure (TDM©), a 31-item questionnaire, which characterizes teams’ stage of development and the degree to which groups have the characteristics of highly effective teamwork in place [70, 71]. The TDM© is ideally suited for Function QUERI’s evaluation of teams because it has been tested in a variety of healthcare settings and maintains psychometric strength when applied to a range of group membership sizes. Items from the TDM© also cluster to reflect the four key dimensions of team function: communication, role clarity, cohesion, and goal and means clarity [70]. This will enable the implementation core to (1) assess baseline and post-implementation changes in dimensions of team function, (2) identify variations in team strengths and weaknesses within and across clinical programs at participating sites, and (3) assess the dimensionality (e.g., one summative measure of team function or multiple measures of team function) and predictive power of team function on implementation and program outcomes to inform future implementation efforts.

The implementation core will supplement the TDM© with additional survey and interview questions identified from the literature on healthcare teams [72]. These additional items describe other important features of teams, including size, composition, and the degree to which team membership spans boundaries (e.g., clinical, professional). We will also describe team communication channels (internal, external), decision-making, and establishment of routines.

*Clinical program characteristics* include the components and processes that each site chooses for program delivery. Each site for STRIDE and iHI-FIVES will be provided with clinical program implementation packages that outline both the core and optional elements of each program. We will track how each site decides to structure their program and also assess other important elements associated with delivering the program such as task complexity and uncertainty, and dependence on other teams or clinical units within the organization.

*Environmental context* is an important consideration in our evaluation. Clinical programs and their teams operate within the broader context of local VAMCs that vary by facility complexity, organizational climate, presence of policies and practices to support innovative practices, and historical performance. These factors may work individually or in combination to support or challenge a team’s ability to accomplish intended goals.

*Implementation outcomes* are the intermediate result of deliberate action in implementing new practices/services [73]. They serve as indicators of implementation processes that are the pre-condition for attainment of intended clinical and system-level change (i.e., program outcomes such as reductions in admission to nursing homes). This distinction is important, as it provides more specificity of the mechanisms underlying implementation successes or failures [73]. Given the overall goals of Function QUERI and the goals of our operational partners, we will focus on three main types of implementation outcomes: penetration, fidelity, and cost. Penetration of clinical programs is generally defined as proportion of eligible patients and/or caregivers who received clinical program services (e.g., referrals to iHI-FIVES). Fidelity will be assessed via measures that describe patient/caregiver engagement (e.g., patient attendance for Group PT, caregivers’ attendance for iHI-FIVES), dose, and adherence to the protocol (e.g., STRIDE participants with at least one supervised walk). To assess budget impacts, we will assess costs related to program implementation and program delivery at each site, as well as patient-level resource utilization.

### Analytic approach

Function QUERI’s evaluation of implementation will focus on examining the impact of teams and the CONNECT implementation strategy, within and across projects. We will use a quantitatively driven simultaneous design (QUAN + *qual)* in which quantitative data constitute the core component and are collected in parallel with qualitative data [74, 75]. These two methods will be used to answer related questions, in that quantitative data will be used to evaluate implementation effectiveness and qualitative data to understand how implementation processes (including team processes) and environmental context related to implementation outcomes. Data integration will involve embedding qualitative process data within the quantitative outcomes data (for example, in a matrix format in which program sites are arranged from high to low penetration) to evaluate the relationship between implementation outcomes and process for both implementation strategies (e.g., REP alone vs. REP + CONNECT).

To assess the relationship between teams and implementation outcomes, we will examine how fidelity and penetration outcomes change over time by team measures. For example, prior work [70] has demonstrated that items from the TDM© reflect team communication, role clarity, cohesion, and goal and means clarity. Survey items from the larger team survey may cluster to reflect other dimensions of team function, which will enable Function QUERI to examine predictive power of team characteristics on implementation and program outcomes. Pre- and post-implementation survey administration will also enable assessment of temporal changes in team function. Specifically, prior experience suggests that baseline team structure and communication effectiveness modifies the impact of CONNECT, with highly functioning or dysfunctional teams receiving less benefit. Thus, by capturing baseline team measures, we will be able to assess for this effect. Furthermore, if we find that the implementation strategy REP + CONNECT improves implementation outcomes compared to REP alone, we will examine whether team measures mediate or moderate this effect following methods of MacKinnon [76] and Kraemer [76, 77].

We anticipate that sites randomized to implementation via REP + CONNECT will achieve higher rates of implementation effectiveness (e.g., higher penetration, fidelity) than sites receiving REP alone. To evaluate the impact of CONNECT on clinical program implementation, penetration, fidelity, and cost outcomes will be assessed only in the post-implementation period and primary analyses will be conducted on implementation outcomes observed in the first post-implementation period to avoid potential confounding with time since implementation. We will use appropriate logistic or linear mixed regression models [78–81], where the main predictor of interest will be REP vs. REP + CONNECT adjusting for clustering of VAMC with either a random effect or by conditioning.

Organization-specific context and processes are likely to affect both implementation and program outcomes. Our quantitative findings will be complemented by additional analyses of context sensitivity and qualitative data from semi-structured interviews with key informants at participating sites. Responses will be coded and analyzed at individual and team levels, using both a priori labels of facility context, team processes and implementation outcomes, and data-derived labels to develop site-level case memo summaries of contextual factors and team processes. From this coded data, we will identify and visually display emergent themes in a matrix, with columns reflecting implementation outcomes (i.e., fidelity and penetration) arranged from high to low, to illustrate patterns in contextual factors and team processes according to implementation outcomes. For example, we will develop a matrix to compare reports of implementation processes and outcomes between REP alone and REP + CONNECT sites. The rows of the matrix will reflect a priori implementation measures, and the columns will reflect whether responses are from REP or REP + CONNECT sites.

Utilizing a similar approach across projects, we will perform budget impact analysis for STRIDE and iHI-FIVES multi-site projects to frame affordability to the VHA [82]. Depending on the evaluation results of STRIDE and iHI-FIVES implementation projects, budget impacts will particularly focus on comparing total costs by the implementation strategy of REP alone vs. REP + CONNECT. Since CONNECT is expected to be more time-intensive and expensive than REP, it is critical to consider the relative gains (if any) to both team function and patient outcomes from adding CONNECT training to REP. For these projects, we will also consider variability in budgetary impact by site (e.g., costs may differ by low versus high penetration sites or by team composition). We will calculate the budget impacts of the clinical programs and compare them to each program’s value. The value will be defined in light of all of the evaluation evidence. For example, the value may be framed as total budget impact per unit gain in patient function or as total budget impact compared to the clinical team’s narrative on how a program benefited patients. We will also consider framing budgetary impact against different domains, such as total costs by site or costs per Veteran participant. The Function QUERI investigators leading the clinical programs will work with the implementation core and VA operational partners to develop appropriate comparisons.

## Discussion

### Limitations and challenges

The Function QUERI set an ambitious and important agenda to explore ways to effectively disseminate and implement innovative evidence-based clinical programs across the VA health system. There is a significant amount of work that presents challenges and opportunities. First, conducting the implementation and evaluation, with an a prior stepped-wedge design in ever changing real-world settings, forces Function QUERI to delicately balance considerations of study design and voluntary participation in implementing clinical programs. To this end, our use of REP to work with sites in a systematic and standardized way is a great strength in facilitating local adaptations to optimize implementation. Second, our selection of volunteer sites may limit heterogeneity in our sample. However, there is a great deal to learn about implementation processes and adaptations in real-world settings to inform continuous improvement and to the likelihood of effective uptake and sustainability of valuable clinical intervention. Third, our measurement approach involves significant effort in primary data collection of patient and provider surveys, interviews, and extraction of secondary administrative/clinical data with attention to common measures across three contextually disparate clinical contexts. The challenges of data collection and management may be outweighed by the opportunity to generate a broader, nuanced understanding of implementation in hospital settings and to facilitating effective interdisciplinary team function across VA settings.

### Summary

Function QUERI will achieve an immediate impact on the VHA by providing access to evidence-based clinical services for a large group of vulnerable Veterans at risk for functional decline and loss of independence. A long-term impact will be to enhance VA’s capacity for clinical innovation through development and testing of an implementation intervention (CONNECT) to enhance uptake of evidence-based programs in interdisciplinary teams. To this end, the Function QUERI program will specify dimensions of team characteristics and function that enhance capacity for clinical innovation and uptake of evidence-based programs. Synthesizing findings within and across projects, we will translate implementation findings to identify the contextual factors and components from CONNECT that improve team processes and function to optimize future wide-scale implementation of VA clinical programs.

## Additional file

Additional file 1: Function QUERI implementation activities, by phase of REP (indicated by green arrows) and highlighting application of CONNECT (red arrows) for each project. (DOCX 222 kb)

## Abbreviations

CFIR: Consolidated Framework for Implementation Research
Group PT: Group physical therapy for knee osteoarthritis
HI-FIVES: Helping invested family members improve veteran experiences study
iHI-FIVES: Implementation of helping invested family members improve veteran experiences study
IoC: Implementation of change
PT: Physical therapy
QUERI: Quality enhancement research initiative
REP: Replicating Effective Programs
STRIDE: Assisted early mobility for hospitalized older veterans
TDM^©^: Team Development Measure
VA: Veterans administration
VAMC: Veterans Administration Medical Center
VHA: Veterans Health Administration
