# Supplementary material for: Supporting teams to optimize function and independence in Veterans: a multi-study program and mixed methods protocol
Source: Implement Sci. 2018 Apr 20;13:58. doi: 10.1186/s13012-018-0748-3 (PMC5910600; doi:10.1186/s13012-018-0748-3)
Supplement: Supplementary file 1 — Function QUERI implementation activities, by phase of REP (indicated by green arrows) and highlighting application of CONNECT (red arrows) for each project. (DOCX 222 kb) [file 13012_2018_748_MOESM1_ESM.docx]

Additional file 1 Function QUERI implementation activities, by Phase of REP (indicated by green arrows) and highlighting application of CONNECT (red arrows) for each project


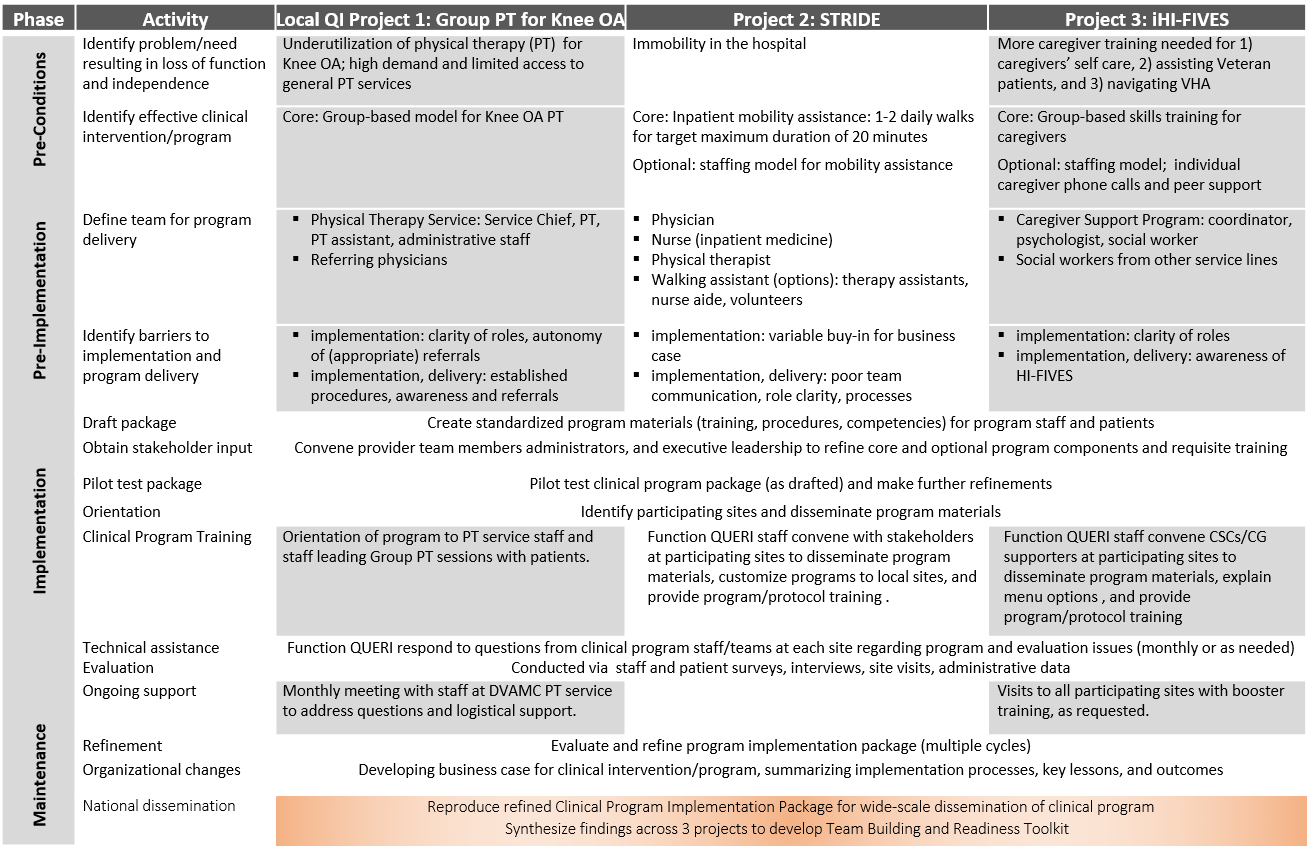


CONNECT

CONNECT

CONNECT
